# Photosensitive inhibition of the GABA system *in vitro*

**DOI:** 10.1038/s41598-020-59915-2

**Published:** 2020-02-21

**Authors:** Lei Sun, Xinying Liu, Xue Li, Mi Li

**Affiliations:** 1 PTN Graduate Program, School of life science, Tsinghua University, Beijing, 100084 China; 2 Shijiazhuang Michen Biological Technology Co., Ltd, Shijiazhuang, 050000 China; 3 School of Medicine, Tsinghua University, Beijing, 100084 China; 4 Institute of Medical research, Northwestern Polytechnical University, Xian, 710072 China

**Keywords:** Cellular neuroscience, Inhibition

## Abstract

In the central nervous system (CNS), γ–aminobutyric acid A (GABA_A_) receptors mediate two types of inhibitory effects. Phasic inhibition involves the activation of synaptic GABA_A_ receptors, and tonic inhibition is mediated by extrasynaptic GABA_A_ receptors. GABA_A_ receptors are important regulators of neuronal activity and are involved in a range of neurological disorders. In this study, we conducted sIPSCs recordings on hippocampal CA1 pyramidal neurons in WT SD rats and found that exposure to blue light could specifically block the tonic inhibition and sIPSCs, and regulate neuronal activity. These observations indicate the existence of a non-opsin photosensitive pathway that regulates the GABA inhibitory system in the CNS.

## Introduction

In the human brain, the principal inhibitory system acts *via* GABA_A_ and GABA_B_ receptors. GABA_A_ receptors mediate two types of inhibition. One is phasic inhibition, which involves activation of the synaptic αβγ GABA receptor subunit assembly, by transient high concentrations of GABA, whereas the second inhibition is tonic inhibition, which depends on the continuous activation of extrasynaptic high affinity GABA_A_ receptors, that include α4, α5, α6, and δ subunits^1,2^. Synaptic GABA_A_ are extensively expressed throughout the brain. Extrasynaptic GABA_A_ receptors are widely expressed in cerebellar granule cells^3^, neocortex^4^, thalamus^5^, the striatum^6^, hypothalamus^7^, and hippocampus^8^.

The balance of excitation and inhibition (E:I) is fundamental for normal brain function. Dysfunction in the GABA inhibitory system is associated with several neurological disorders, such as depression, stress, and epilepsy. Detailed investigation of the GABA inhibitory system in research primarily depends on the use of GABA receptor antagonists or optogenetic methods. A recent study reported that the activity of fast-spiking interneurons in the cerebral cortex could be suppressed by heat produced by sustained light exposure with a power of 3 mW~15 mW, which is commonly used in optogenetic procedures. However, this phenomenon was not observed in the hippocampal CA1 area^9^.

In this study, we found that blue light could specifically and reversibly block tonic inhibition and sIPSCs in hippocampal CA1 pyramidal neurons in 6- to 8-week-old female WT SD rats. This effect was unrelated to the heating effect, which indicated the existence of a non-opsin photosensitive pathway that regulates the GABA inhibitory system.

## Results

### Blue light inhibited the amplitude and frequency of sIPSCs in hippocampal CA1 pyramidal neurons

**Figure 1:**
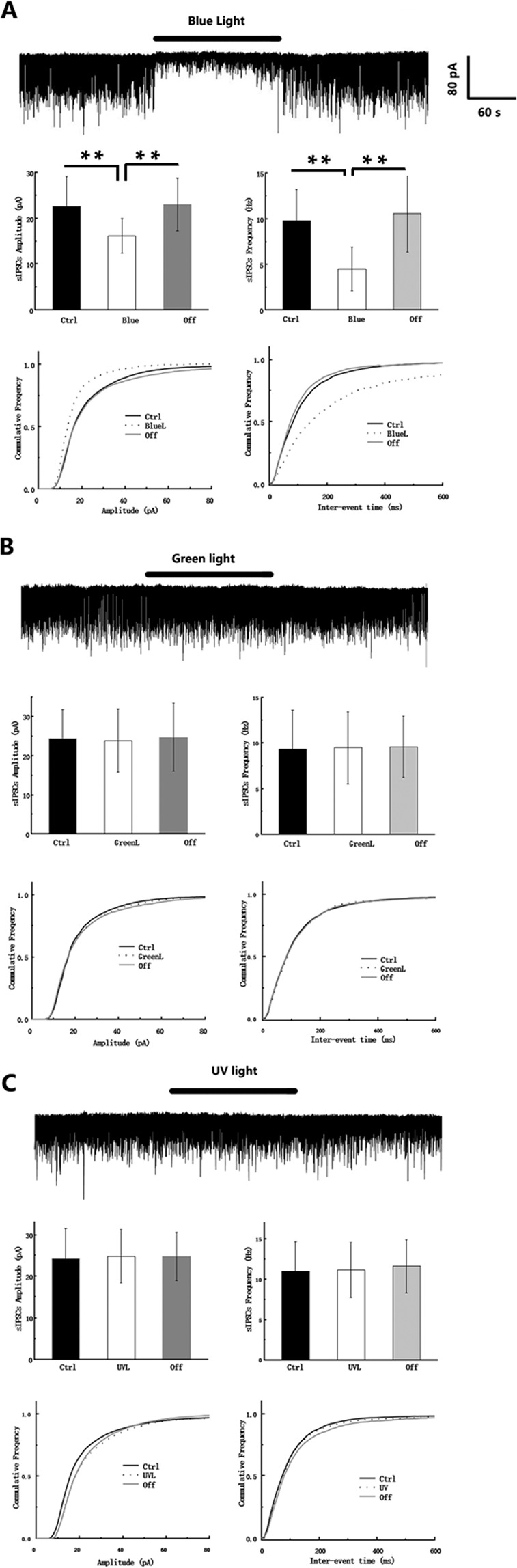
Blue light inhibits sIPSCs. (**A**) Representative traces of sIPSCs in hippocampal CA1 pyramidal neurons in the presence of CNQX (20 μM) and APV (50 μM) with the application of 480 nm blue light (upper). Bar graphs of sIPSCs amplitude and frequency (middle). Blue light significantly decreased sIPSC frequency and amplitude and recovered after the light was turned off. The accumulative amplitude was left-shifted, and the accumulative frequency of inter-events interval was right-shifted with the application (p < 0.001, Kolmogorov-Smirnov test, lower). (**B**) Representative traces of sIPSCs with the application of 580 nm light (upper), bar graphs (middle), and cumulative sIPSC amplitude and frequency (lower) with no differences observed. (**C**) Representative traces of sIPSCs with the application of 360 nm light (upper), bar graphs (middle), and accumulative sIPSC amplitude and frequency (lower) with no differences observed.

A recent study reported that the activity of fast-spiking interneurons in layer V of the cortex could be suppressed by heat produced by exposure to light. However, hippocampal CA1 pyramidal neurons were not affected. In our research, we recorded sIPSCs in hippocampal CA1 pyramidal neurons using 6- to 8-week-old female WT SD rats. A constant blue light was delivered using a mercury light source with a FITC filter. Surprisingly, we found that this exposure induced continuous hyperpolarization (3.71 ± 1.47 mV). We also observed inhibition of the amplitude (ctrl: 22.70 ± 6.42 pA; Blue: 16.16 ± 3.47 pA: off: 23.05 ± 8.73 pA) and frequency (ctrl: 9.80 ± 3.48 pA; Blue: 4.31 ± 2.43 pA: off: 10.62 ± 4.23 pA) of the sIPSCs (Fig. 1A). To investigate whether this effect was wavelength specific, we also tested the effect of UV and green light, both of which had no effect (p > 0.05), even with longer exposure times (Fig. 1B,C). This result indicates the possibility that there is a specific, blue light sensitive pathway and that this pathway is capable of regulating neuronal activity in the hippocampus. To test whether this effect was dependent on the frequency of the blue light, we delivered blue light at a variety of frequencies and powers (>10 mW), using a 480 nm laser generator. We observed a relationship between the minimum frequency and power that were required to induce this effect when a 2 ms fixed pulse width was used. Therefore, the induction of this effect was not frequency dependent. However, it required a level of effective illumination power that is associated with most photosensitive proteins (data not shown).

### sIPSCs inhibition was unrelated to the heating effect of light

**Figure 2:**
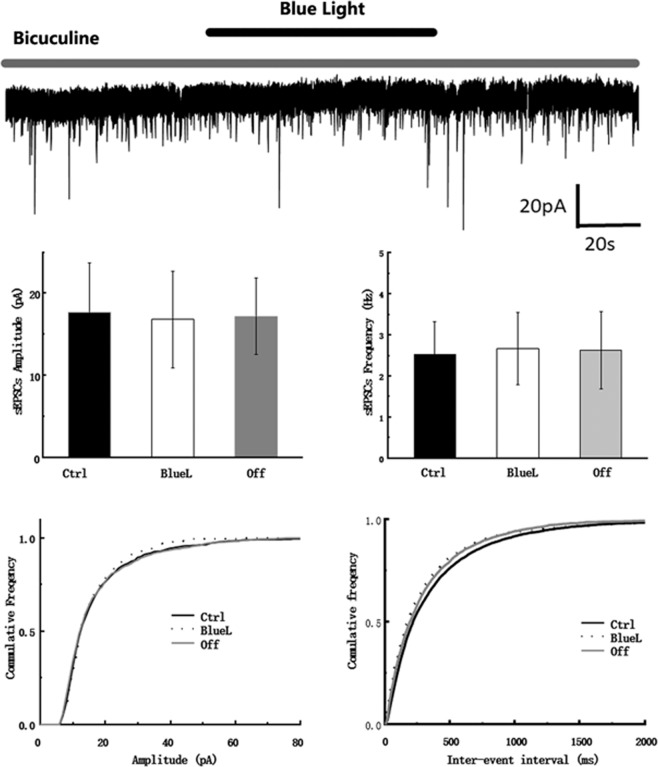
GABA_A_ receptor antagonist, bicuculine, blocked the effect of 480 nm blue light. Representative traces of tonic current inhibition by 10uM bicuculine. Exposure to blue light showed no effect because the GABA_A_ receptors were blocked by bicuculine (upper). The bar graphs (middle) and the accumulative sEPSC amplitude and frequency with the 480 nm application light (lower) showed no differences.

From an evolutionary point of view, it is not necessary to express photosensitive proteins on the membranes of hippocampal CA1 pyramidal neurons in the brain, as these neurons do not receive any natural light. In contrast, light-induced heat has been reported to impair ion channel conductance^10^, synaptic transmission^11,12^ and spiking^13^ across various brain area as observed using recordings on slice. Therefore, we tried to determine whether this effect was directly induced by light or by its heating effect. We compared the heating effect of UV, blue and green lights using a micro thermoprobe. Without perfusion, a temperature increase of 0.3 °C was detected within 60 s, (Fig. 2A), while with perfusion an increase of 0.1 °C was observed (data not shown). Moreover, when we increased the temperature of the perfusion solution by 0.1 °C ~1 °C, the holding current, frequency or amplitude of sIPSCs remained unchanged (Fig. 2B). This indicated that the inhibition of tonic currents and IPSCs is unrelated to the heating effect of light. Therefore, we concluded that this pathway is photosensitive but not thermosensitive.

### Exposure to blue light inhibited the firing rate

**Figure 3:**
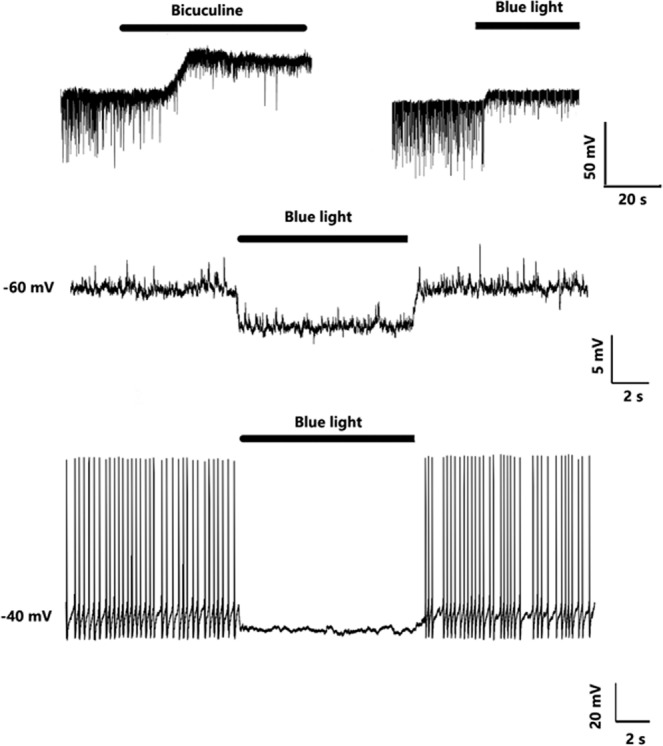
Blue light hyperpolarize the membrane potential and inhibit the formation of action potential. GABA_A_ mediated tonic currents were inhibited by 10uM bicuculine and blue light (upper). Membrane potential hyperpolarization was induced by exposure to blue light under current clamp and without current injection (middle). The neuronal firing was blocked by exposure to blue light when current was injected to depolarize the membrane potential to the threshold (lower).

To confirm the effect of blue light under current clamp recording, as expected, exposure to blue light induced a slight hyperpolarization (Fig. 3A,B). To explore whether this hyperpolarization impaired neuronal activity, we injected current and observed that exposure to blue light did not have any effect when the baseline of membrane potential was approximately −70 mv, which was relatively far from the threshold. However, when the baseline just exceeded the threshold, exposure to blue light clearly suppressed the firing rate (Fig. 3C).

### Specific inhibition of the GABA system

**Figure 4:**
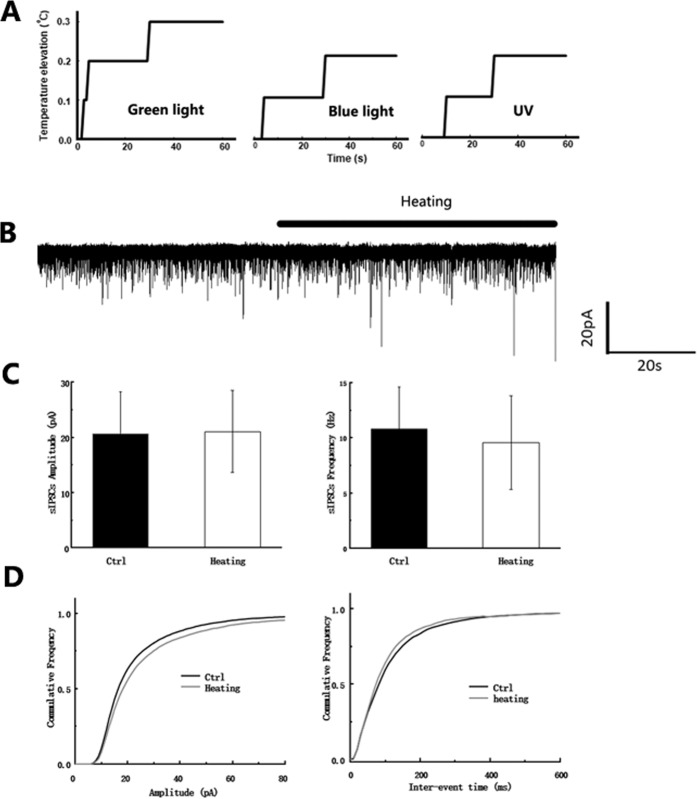
The influence of temperature on the tonic currents and sIPSCs. (**A**) Heating effect of 480 nm blue light, 580 nm light and 360 nm light (upper) with exposure of 1 min. (**B**) The influence of temperature on the tonic currents and sIPSCs. (left panel). (**C**) Bar graphs (down) and (**D**) cumulative sIPSCs amplitude and frequency of blue light application (down) has no difference observed.

Then we tested whether this was a general effect or if it was specific to the regulation of the GABA inhibitory system. The GABA_A_ antagonists, bicuculine and gabazine, were applied before exposure to blue light. As expected, the effect induced by blue light exposure alone was not observed (p > 0.05, Fig. 4). This observation indicated that exposure to blue light did not directly impair the activity of hippocampal CA1 neurons. Moreover, based on the specific internal solution that we used, we were able to reverse this effect when the membrane potential was held in a range between −30 mV or 0 mV, respectively. This is similar to the equilibrium potential of Cl^−^, which suggests that this effect was mediated by Cl^−^ channels (data not shown). These results confirmed the specific inhibition of the GABAergic system with exposure to blue light.

Lastly, this inhibition of GABA system mediated by blue light could be observed in the paraventricular nucleus (data not shown). This indicates it might be a general phenomenon in the brain.

## Discussion

A recent study reported that gene transcription and neuronal firing rates were impaired following exposure to blue light^9,13^. However, the underlying mechanism was thought to be due to the heat that was produced by light exposure. Our results proved that blue light directly interacted with GABAA receptors.

GABA_A_ receptors are broadly expressed in several areas of the brain and are potential targets for the treatment for a range of neuronal diseases. Currently, it is common to use pharmacological or optogenetic manipulations to study the activity of the GABA system in clinical and experimental research. Our results provide a new method to modulate the activity of the GABA system. However, in different brain regions, the GABA_A_ subunits responsible for tonic inhibition were different. Thus, even if this effect was confirmed in the paraventricular nucleus of the hypothalamus, further work is required to confirm this effect in other brain regions.

480 nm blue light is widely used in neuroscience research to turn on or off channelrhodopsin-expressing neurons. Thus, exposure to blue light might induce unwanted side effects. A recent study reported that the heating effect of light might inhibit the firing rate of MSN neurons through HCN channels. However, this phenomenon was not observed in the hippocampus. This indicates that there may be a number of direct and indirect pathways in the brain that regulate neuronal activity. Our results showed that the induced hyperpolarization by blue light was much smaller than that produced by bicuculine. It also demonstrated that the modulation of the firing rate was only produced when the membrane potential passed the threshold. This raises the question of whether this could have consequences *in vivo*. The answer to this question remains to be explored.

## Methods

### Slice preparation

All experiments were performed on brain slices that were obtained from 6- to 8-week-old female SD rats. All procedures were carried out with the approval of the Tsinghua University Institutional Animal Care and Use Committee, with the Animal Welfare Assurance Identification number, F60-00228; A5061-01.All animal use in this research complied with the humane use of animals in laboratory research as set out by international guidelines In brief, the brain was rapidly removed and placed in an ice-cold slicing solution, composed of 225 mM sucrose, 2.5 mM KCl, 0.5 mM CaCl_2_, 7 mM MgCl_2_, 1.25 mM NaH_2_PO_4_, 26 mM NaHCO_3_, and 10 mM glucose. The solution pH was adjusted to 7.4 after bubbling with 95% O_2_ and 5% CO_2_ for 30 minutes. Horizontal slices (350 μm thick) were cut at the level of the hippocampus using a Leica 1200 s vibrating blade microtome (Wetzlar, Germany). The slices were then incubated at 34 °C for 30 min in normal artificial cerebrospinal fluid (ACSF) containing 120 mM NaCl, 2.5 mM KCl, 0.5 mM CaCl_2_, 7 mM MgCl_2_, 1.25 mM NaH_2_PO_4_, 26 mM NaHCO_3_, and 10 mM glucose. The slices were maintained at room temperature in the above solution and bubbled with 95% O_2_/5% CO_2_ until required.

### Recording procedures and analysis

During recording, the slices were continuously perfused with normal ACSF at physiological temperatures (32–35 °C). Whole-cell recordings were performed in hippocampal CA1 pyramidal neurons using an EPC-10 USB amplifier and PATCHMASTER software (HEKA Eletronik), as previously described^14^. The slices were viewed using a Scientifica sliceScope (Uckfield, England), equipped with differential interference contrast-infrared optics. The membrane potential was voltage-clamped at −70 mV, and the signals were filtered at 2.9 KHz. The recording electrodes were pulled from thick-walled borosilicate glass (BF-150-86-10; Sutter, Novato, US) and had a resistance of 3–5 MΩ. The series resistance was less than 20 MΩ and was compensated to 70~90%. Two types of intracellular solutions were used for sIPSCs recording. One was a high Cl^−^ internal solution containing 135 mM KCl, 10 mM HEPES, 0.5 mM EGTA, 0.3 mM Na-GTP, 4 mM Mg-ATP, and 10 mM Na_2_-phosphocreatine. The second solution was a medium Cl^−^ internal solution containing 85 mM K-gluconate, 50 mM KCl, 10 mM HEPES, 0.5 mM EGTA, 0.3 mM Na-GTP_2_, 4 mM Mg-ATP, and 10 mM Na_2_-phosphocreatine. The pH was adjusted to 7.2 with KOH.

For sIPSCs, 20 µM CNQX (Sigma St. Louis, MO) and 50 µM APV (Sigma, St. Louis, MO) were added to the recording bath. Each sIPSCs event was fully characterized based on the parameters of amplitude, frequency, half-width, and charges. The MiniAnalysis software (Synaptosoft, Inc., Leonia, NJ) was used to detect and analyze these parameters. After the software selected individual events, each detected event was visually inspected to eliminate artifacts. To measure the tonic currents, 100 µM bicuculine (Sigma St. Louis, MO) was added to the recording bath.

The ultraviolet, blue, and green lights were produced by a mercury lamp carrying DAPI (EX: 377/50, DM: 409, BA: 447/60 BP), FITC(EX: 482/35, DM: 506, BA: 536/40 BP), and mcherry(EX: 562/40, DM: 593, BA: 641/75)filters. The blue light that was used to check the frequency of the dependent effect came from a 480 nm laser source (Laser&Optic Century co., Ltd, Shanghai, China). For sIPSCs recordings, we decreased the strength of the illumination from all three colors to maintain the recordings, as there was a loss of the patch with strong UV illumination.

### Statistics

The statistics were calculated using functions provided in GraphPad Prism 7 for Windows (GraphPad Software Inc., La Jolla, CA). All data were expressed as means ± standard deviation (SD). The significance was set at p < 0.05. The statistical significance was assessed using the paired Student’s t-test or random block variance analysis and corrected using Welch’s method that assesses variance inequalities.
